# Employment conditions and use of gastric cancer screening services in Korea: a nationwide survey

**DOI:** 10.1186/s12889-019-6841-y

**Published:** 2019-05-02

**Authors:** Hye-Young Shim, Jae Kwan Jun, Ji-Yeon Shin

**Affiliations:** 1 Department of Preventive Medicine, School of Medicine, Eulji University, Daejeon, Republic of Korea; 2 Department of Rehabilitation Medicine, Seoul National University Bundang Hospital, Seoul National University College of Medicine, Seongnam, Republic of Korea; 3 National Cancer Control Institute, National Cancer Center, Goyang, Republic of Korea; 4 Department of Preventive Medicine, School of Medicine, Kyungpook National University, Daegu, Republic of Korea

**Keywords:** Employment status, Precarious employment, Preventive health services, Cancer screening, Gastric Cancer

## Abstract

**Background:**

Although it is well known that employment conditions exert considerable effects on health and health equity, the association between employment conditions and the use of preventative health services has rarely been studied. We explored whether inequities in the use of preventative services were associated with employment conditions. We used gastric cancer screening as a surrogate for the use of preventative health services.

**Methods:**

The study population was derived from the Korea National Health and Nutrition Survey IV (2007–2009), which included data on 5626 individuals over 40 years of age. Employment conditions were grouped by employment status, work hours, employment contract term, and salary source. Participants who had undergone gastroscopy or an upper gastrointestinal series within the past 2 years were considered to have used cancer screening services according to the National Cancer Screening Program guidelines. Odds ratios (ORs) and 95% confidence intervals (CIs) were estimated using multiple logistic regression analysis. As the survey procedure incorporated sample weights, we adjusted our calculations to consider the complex sample design.

**Results:**

Self-employed workers were less likely to participate in regular cancer screening than were wage workers (OR = 0.79, 95% CI = 0.68–0.92), and part-time workers were less likely to participate than were full-time workers (OR = 0.81, 95% CI = 0.67–0.99). Among wage workers, temporary workers and daily workers exhibited lower participation rates than did regular workers (OR = 0.81, 95% CI = 0.63–1.05 and OR = 0.58, 95% CI = 0.44–0.76, respectively). Dispatched workers also exhibited lower participation rates (OR = 0.45, 95% CI = 0.25–0.80).

**Conclusions:**

We found obvious inequities in the use of preventative health services associated with various employment conditions. Self-employed, irregular, and dispatched workers were significantly less likely to participate in cancer screening than were other workers. Political efforts should be made to reduce employment insecurity and to improve participation in preventative screening services by vulnerable employees so as to resolve the evident health inequities.

## Background

Employment status and employment conditions exert considerable effects on health and health equity [1]. Several employment-related conditions, including precarious work (informal, temporary, or contract work), were reportedly associated with poor health status [2–4] and health inequities. Job insecurity has been found to decrease perceived health [5], increase psychological distress [6], and lead to poor physical health [7]. The evidence even indicates that mortality is significantly higher among precarious workers than among permanent workers [8].

Over the last few decades, in the name of neo-liberalism, the deregulation of labor markets, increased competition, forced restructuring or downsizing, and privatization have become commonplace globally [9]. As a result, precarious work and job instability have increased worldwide, and in South Korea, following the 1997 financial crisis, the level of unstable employment also increased [10] in association with changes in workers’ health [11–14].

Equal access to preventative healthcare has been emphasized as a public health priority in the “Health For All” agenda set out in the Alma-Ata declaration of the World Health Organization in 1978 [15]. Nonetheless, previous research revealed that socioeconomic inequalities were evident in terms of the use of both healthcare and preventative health services [15, 16]. In most cases, those of high socioeconomic position (SEP) used preventative services more than did low-SEP groups [15, 16]. However, few studies have focused on employment conditions, despite the fact that these are important components of individual SEP. Employment conditions (including precariousness) can interact with various socioeconomic factors throughout the lifespan and across the health spectrum (i.e., disease prevention, diagnosis, the quality of care, the chances of survival, and the consequences of ill-health) and may thus give rise to health inequalities.

In the present study, we explored whether inequity in the use of preventative services was related to employment conditions using data from a nationally representative survey. We hypothesized that employment conditions, especially employment precariousness influences the use of preventative health activities.

We focused on cancer screening (as did many previous studies) as a surrogate for the association between socioeconomic inequality and the use of preventative services [15, 17, 18]. In Korea, the government has operated a National Cancer Screening Program (NCSP) since 2005, providing Medicaid enrollees and National Health Insurance (NHI) beneficiaries in the lower half of the income strata with free screening for five major types of cancer (stomach, breast, cervical, colorectal, and liver). NHI beneficiaries in the upper half of the income strata receive screening services for the same types of cancer from the NHI Corporation, and are required to pay only 10% of the cost [19]. Of the five cancers, breast and cervical cancer screenings are restricted to females; colorectal cancer screening is provided for those aged ≥50 years, and liver cancer screening is restricted to high-risk groups (those with chronic hepatitis as determined by serological evidence of infection with hepatitis B or C virus, or liver cirrhosis) aged ≥40 years. Gastric cancer screening is provided for both males and females aged ≥40 years [19]. Thus, considering that gastric cancer screening is provided for both sexes and is targeted to relatively young people (in their working years), this was the most appropriate surrogate of the five screening services when examining a possible association between employment conditions and the use of preventative services. Moreover, as gastric cancer screening was either the first or second most commonly used screening service of the five available services from 2004 to 2013 [19], we expected that it could include the widest range of people and would not be limited by sex, age, and high risk conditions.

Therefore, using gastric cancer screening as a surrogate for the use of preventative health services, we explored whether such use was associated with employment conditions. These comparisons afforded useful insights into inequities in the use of such services.

## Methods

### Study design and population

We analyzed data from the fourth Korea National Health and Nutrition Examination Survey (KNHANES IV, 2007–2009), which is an ongoing, multicomponent, nationally representative survey of the non-institutionalized Korean population conducted by the Korea Centers for Disease Control and Prevention (KCDC). The survey employs a stratified, multistage, probability sampling design; the sampling units are taken from household registries with reference to sex, region, and age group. In 2007, the KNHANES adopted a rolling sample design; the dataset for KNHANES IV (2007–2009), which was used in the present study, included 200 sampling units from the South Korean target population that were selected at random from the primary sampling units. Next, the sampling units were used to identify 23 households per sample in each year, yielding a total of 4600 households.

**Fig. 1:**
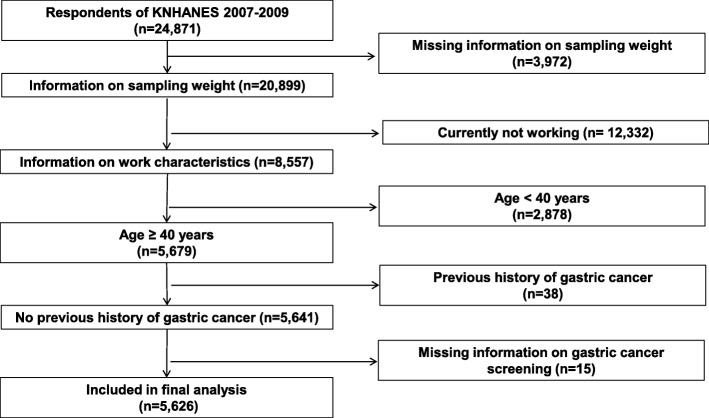
Schematic diagram depicting the study population

The KNHANES assessments include a health interview, a health examination, and a nutritional survey; we used data from the health interview to identify sociodemographic and employment characteristics and cancer screening behaviors. The details of the survey have been described elsewhere [20]. The survey was approved by the institutional review board (IRB) of the KCDC in 2007–2009 (approval nos. 2007-02CON-04-P, 2008-04EXP-01-C, and 2009-01CON-03-2C). Of the 24,871 individuals who participated in KNHANES IV, those for whom sampling weights were missing were excluded (*n* = 3972), as were those not currently working (*n* = 12,332), those not eligible for gastric cancer screening (< 40 years of age; *n* = 2878), those with a previous history of gastric cancer (*n* = 38), and those who did not answer the question about gastric cancer screening (*n* = 15). Ultimately, data from 5626 individuals over 40 years of age were selected for the final analysis (Fig. 1).

### Measures

The NCSP recommends that subjects aged ≥40 years should undergo gastric cancer screening on a biennial basis via either upper endoscopy or the taking of an upper gastrointestinal series (UGIS) [19]. Participation in gastric cancer screening was assessed by asking the following question: “When was the last time you had a gastric cancer screening examination (endoscopic gastroscopy, UGIS, or endoscopy + UGIS)?” The possible responses included less than 1 year ago, 1–2 years ago, more than 2 years ago, and never. For the purposes of the present study, participants who had undergone gastroscopy or a UGIS within the previous 2 years were considered to be “participants obeying the recommendations” of the NCSP guidelines.

Among participants who were currently working, employment status was classified by asking the following question: “Which of the following best describes your work?” The possible answers included wage worker, self-employed, and unpaid family worker. Participants were asked in detail about their occupation and then classified into six categories based on the Korean Standard Classification of Occupations [21]: professional/manager/administrator, office worker/clerk, sales/service worker, agricultural/fishery worker, plant/machine operator, and manual worker. Participants were also asked about the industries in which they worked, with the possible answers including agriculture/fishery/mining, manufacturing, construction, and other services. The number of work hours per week was obtained by asking the following question: “How much time do you spend working in a workplace during a 1-week period, including overtime?” The answers were written as Arabic numbers and classified as ≤40, 41–59, and ≥ 60 h per week. The work schedule was explored by asking the following the question: “Do you mainly work during the day (6:00 A.M. to 6:00 P.M.) or during different times (including night work and shift work)?” The possible answers included mainly working in the day and shift work. Work type was assessed in terms of the number of working hours by the following question: “Which of the following describes your working hours?” The possible answers included full time and part time.

Wage workers were further classified by (1) employment contract term and job security and (2) salary source and business command. In terms of employment contract term and job security, a wage worker was classified as either regular, temporary, or daily worker. Those with more than 1 year of employment contract or those who were admitted to the company according to the set employment procedure and received various benefits such as compensation and severance payments were defined as “regular workers.” Those with less than 1 year of employment contract or those without a fixed employment contract who were hired out of necessity of completing a business for more than 1 month and less than 1 year were defined as “temporary workers”. Those with less than 1 month’s employment contract with an individual, a household, or a business, or those who were employed on a daily basis and were paid day-to-day for their work were defined as “daily workers.” When classifying wage workers in terms of salary source and business command, a wage worker was classified as one of the following: worker working in original company, a dispatched worker, or an outsourced worker. Those whose salary and employment relationship is controlled by the dispatched business owner, but the job command and order were controlled by the currently working business owner were defined as “dispatched workers.” On the other hand, those who were commanded and supervised by the outsourced business owner in all matters, such as wage, employment status, and job command and order were defined as “outsourced workers”.

As in previous studies investigating the factors associated with cancer screening [22–24], we included age, gender, educational level, monthly household income, marital status, and health-related behavioral factors (smoking status and alcohol consumption) as covariates. Age was categorized as 40–49, 50–59, 60–69, or ≥ 70 years; educational level was classified as less than middle school, middle or high school, or college or above; marital status was classified as married or not married (single, divorced, and widowed); and income was classified as < 1000 USD, 1000–3000 USD, and ≥ 3000 USD per month per household. Alcohol consumption was divided into three categories: non-binge drinkers (a non-drinker or social drinker who reported binge drinking no more than once per month), binge drinkers (binge drinking 1–4 times per month), and frequent binge drinkers (binge drinking more than twice per week) [22]. Smoking status was categorized into three groups: never-smoker (has never smoked), ex-smoker (has quit smoking), and current smoker (smokes daily or intermittently).

### Statistical analysis

All statistical analyses were performed using the SAS software package (ver. 9.4, SAS Institute, Cary, NC, USA). The survey procedure was adjusted (according to the KCDC guidelines) to reflect the complex survey design, and included appropriate sampling weights to obtain accurate estimates representative of the non-institutionalized Korean population. Descriptive analyses were used to assess cancer screening status by baseline characteristics, and the chi-squared test was employed to compare groups in terms of categorical variables. Next, odds ratios (ORs) and 95% confidence intervals (CIs) were estimated using multiple logistic regression analysis incorporating sample weights, and the figures were adjusted to reflect the complex sample design of the survey. To investigate that employment condition is still associated with cancer screening after step by step adjustment of covariates, three models were constructed to obtain adjusted ORs: model 1 adjusts for age and gender, model 2 additionally adjusts for educational level and monthly household income, and model 3 additionally adjusts for health-related behaviors (smoking and alcohol consumption).

## Results

Of the 5626 currently working individuals assessed, 61.3% were males and 38.7% females, their mean age was 54.9 years, and 46.7% (*n* = 2626) reported that they had undergone screening for gastric cancer in the previous 2 years. In terms of employment status, wage workers comprised 51.2% of all current workers, and this group had a higher screening rate for gastric cancer than did the self-employed and unpaid family workers. In terms of industry, workers in construction were less likely to participate in regular gastric cancer screening. Part-time workers comprised 14.4% of all working participants and had a lower cancer screening participation rate than did full-time workers. Regular workers comprised 64.5% of all wage workers, and this group had higher screening rates than did temporary and daily workers (48.4 and 38.9%, respectively). Dispatched or outsourced workers comprised about 13% of all wage workers and had much lower participation rates for gastric cancer screening than did workers working in their original companies (Table 1).

**Table 1:** Characteristics of study participants by cancer screening status (*n* = 5626)

Variable	n	Weighted %^c^	Regular gastric cancer screening		*P-*value
			Yes (*n* = 2626)	No (*n* = 3000)	
			Weighted %^c^	Weighted %^c^	
Age (years)					
40–49	2125	49.7	43.3	56.7	< 0.001
50–59	1663	31.8	51.4	48.6	
60–69	1204	13.3	50.3	49.7	
≥ 70	634	5.2	39.0	61.0	
Gender					
Male	2878	61.3	47.0	53.0	0.977
Female	2748	38.7	46.3	53.7	
Educational level^a^					
Below middle school	2066	25.5	45.8	54.2	< 0.001
Middle or high school	2577	51.8	45.4	54.6	
College or above	982	22.7	51.9	48.1	
Marital status^a^					
Married	4782	87.4	48.1	51.9	< 0.001
Not married (single, divorced, widowed)	819	12.6	38.3	61.7	
Monthly household income^a^					
< 1000 USD	1324	14.7	42.2	57.8	< 0.001
1000–3000 USD	2174	40.6	45.6	54.4	
≥ 3000 USD	2039	44.6	51.1	48.9	
Alcohol consumption					
Non-binge drinker	3724	59.0	46.9	53.1	0.007
Binge drinker	1504	32.2	47.7	52.3	
Frequent binge drinker	398	8.8	40.7	59.3	
Smoking^a^					
Never	3045	45.3	47.5	52.5	< 0.001
Ex-smoker	1303	26.7	50.6	49.4	
Current smoker	1274	27.9	40.6	59.4	
Employment status^a^					
Wage worker	2505	51.2	48.9	51.1	0.001
Self-employed	2511	42.0	43.2	56.8	
Unpaid family worker	598	6.8	52.3	47.7	
Occupational type^a^					
Professional/manager/ administrator	684	15.7	52.5	47.5	< 0.001
Office worker/clerk	382	8.8	55.2	44.8	
Sales/service worker	1147	23.1	40.8	59.2	
Agricultural/fishery worker	1470	13.7	49.5	50.5	
Plant/machine operator	860	20.7	42.0	58.0	
Manual worker	1077	17.9	46.0	54.0	
Industry^a^					
Agriculture/fishery/ mining	1745	18.0	49.6	50.4	< 0.001
Manufacturing	590	13.5	52.2	47.8	
Construction	368	9.6	36.7	63.3	
Other services	2868	58.9	45.1	54.9	
Work hours (/week)^a^					
≤ 40	2400	41.6	46.3	53.7	0.109
41–59	1732	32.2	49.8	50.2	
≥ 60	1446	26.2	43.9	56.1	
Work schedule^a^					
Day work	4757	82.6	47.2	52.8	0.290
Shift work	837	17.4	43.9	56.2	
Work type^a^					
Full time	4758	85.6	47.4	52.6	0.022
Part time	844	14.4	43.4	56.6	
Wage workers(*n* = 2505)					
Employment status by employment contract term and job security^b^					
Regular worker	1506	64.5	52.2	47.8	< 0.001
Temporary worker	498	17.6	48.4	51.6	
Daily worker	483	17.9	38.9	61.1	
Employment status by salary source and business command^b^					
Working in original company	2155	87.1	50.5	49.5	0.001
Dispatched worker	76	3.2	32.9	67.1	
Outsourced worker	249	9.7	43.0	57.0	

^a^ The totals do not equal 5626 because of missing data

^b^ Measured only in the wage workers (*n* = 2505), and totals do not equal 2505 because of missing data

^c^ Weighted % was generated using the SAS SURVEYFREQ procedure, to account for weighting and clustering

Table 2 shows the associations between employment conditions and participation in gastric cancer screening within the previous 2 years (in line with NCSP recommendations), based on the multivariable analysis. In terms of employment status, self-employed workers were less likely to participate in gastric cancer screening than were wage workers (Model 3, OR = 0.79, 95% CI = 0.68–0.92). In terms of occupational type, office workers were more likely to participate in gastric cancer screening than were all other workers; in particular, this group significantly differed from sales and service workers, plant and machine operators, and manual workers after adjusting for other variables (Model 3, OR = 0.62, 95% CI = 0.47–0.8; OR = 0.67, 95% CI = 0.50–0.91; and OR = 0.74, 95% CI = 0.55–0.99; respectively). In terms of work type, part-time workers were less likely to participate in regular gastric cancer screening compared with full-time workers (Model 3, OR = 0.81, 95% CI = 0.67–0.99). In terms of work schedule, shift workers were somewhat less likely to participate in regular gastric cancer screening compared with day workers (Model 1, OR = 0.82, 95% CI 0.70–0.97). However, after further adjustment, the difference was not statistically significant.

**Table 2:** Odds ratios (ORs) and 95% confidence intervals (CIs) for participation in gastric cancer screening within the previous two years (in line with NCSP recommendations) by employment conditions (*n* = 5626)

Variable	Model 1^a^		Model 2^b^		Model 3^c^	
	OR	95% CI	OR	95% CI	OR	95% CI
Employment status						
Wage worker	1.00	Reference	1.00	Reference	1.00	Reference
Self-employed worker	0.77	0.67–0.89	0.78	0.67–0.91	0.79	0.68–0.92
Unpaid family worker	1.00	0.78–1.28	1.03	0.80–1.33	1.03	0.80–1.34
Occupational type						
Office worker/clerk	1.00	Reference	1.00	Reference	1.00	Reference
Professional/manager/administrator	0.84	0.63–1.13	0.80	0.60–1.06	0.82	0.61–1.09
Sales/service worker	0.54	0.41–0.71	0.59	0.45–0.77	0.62	0.47–0.81
Agricultural/fishery worker	0.75	0.55–1.00	0.92	0.67–1.27	0.97	0.71–1.34
Plant/machine operator	0.55	0.42–0.73	0.64	0.48–0.86	0.67	0.50–0.91
Manual worker	0.58	0.44–0.77	0.70	0.52–0.95	0.74	0.55–0.99
Work type						
Full-time worker	1.00	Reference	1.00	Reference	1.00	Reference
Part-time worker	0.80	0.66–0.97	0.81	0.67–0.99	0.81	0.67–0.99
Work hours (/week)						
≤ 40	1.00	Reference	1.00	Reference	1.00	Reference
41–59	1.09	0.93–1.27	1.10	0.94–1.29	1.09	0.93–1.29
≥ 60	0.88	0.74–1.05	0.93	0.78–1.11	0.93	0.78–1.11
Work schedule						
Day worker	1.00	Reference	1.00	Reference	1.00	Reference
Shift worker	0.82	0.70–0.97	0.86	0.72–1.02	0.87	0.73–1.04

^a^Model 1: Adjusted for age and gender

^b^Model 2: Additionally adjusted for educational level and monthly household income

^c^Model 3: Additionally adjusted for health-related behaviors (smoking and alcohol consumption)

The associations between employment status and participation in gastric cancer screening by wage workers are shown in Table 3. In terms of employment status by employment contract term and job security, daily workers were less likely to participate in cancer screening than were regular workers, even after adjusting for age, gender, educational level, monthly household income, and health-related behaviors (Model 3, OR = 0.72, 95% CI = 0.53–0.97). Additionally, temporary workers were somewhat less likely to participate in cancer screening than were regular workers, even after adjusting for age and gender (Model 1, OR = 0.81, 95% CI = 0.63–1.05); however, the difference was not statistically significant. In terms of employment status by salary source and business command, dispatched and outsourced workers exhibited lower participation rates in regular gastric cancer screening programs than did workers who worked at their original companies. In particular, dispatched workers were much less likely to participate in such screening than were those working at their original companies and outsourced workers (Model 3, OR = 0.48, 95% CI = 0.26–0.89).

**Table 3:** Odds ratios (ORs) and 95% confidence intervals (CIs) for participation in gastric cancer screening within the previous two years (in line with NCSP recommendations) by employment status among wage workers (*n* = 2505)

Variable	Model 1^a^		Model 2^b^		Model 3^c^	
	OR	95% CI	OR	95% CI	OR	95% CI
Employment status by employment contract term and job security						
Regular worker	1.00	Reference	1.00	Reference	1.00	Reference
Temporary worker	0.81	0.63–1.05	0.94	0.72–1.23	0.96	0.74–1.26
Daily worker	0.58	0.44–0.76	0.70	0.52–0.94	0.72	0.53–0.97
Employment status by salary source and business command						
Working in original company	1.00	Reference	1.00	Reference	1.00	Reference
Dispatched worker	0.45	0.25–0.80	0.48	0.26–0.87	0.48	0.26–0.89
Outsourced worker	0.66	0.49–0.90	0.74	0.54–1.01	0.74	0.54–1.01

^a^Model 1: Adjusted for age and gender

^b^Model 2: Additionally adjusted for educational level and monthly household income

^c^Model 3: Additionally adjusted for health-related behaviors (smoking and alcohol consumption)

## Discussion

### Principal findings

We found that individual employment conditions were associated with the use of preventative health services such as gastric cancer screening. Self-employed and precarious workers such as temporary/daily workers and dispatched/outsourced workers exhibited lower participation rates in cancer screening than did wage workers, regular workers, and workers working in their original companies. These findings suggest that the observed disparities in equity may reflect barriers to accessing preventative healthcare services imposed by employment conditions.

The number of precarious workers is increasing worldwide, and this trend is also evident in Korea [25]. The irregular workforce of South Korea comprises 22.3% of the entire workforce, which places the country fifth in the world in terms of the percentage of such workers. Indeed, the Korean average of 22.3% is higher than the 11.3% average for all countries in the Organization for Economic Co-operation and Development (OECD) in 2015 [26]. Additionally, the proportion of self-employed participants in the Korean labor market is very high compared with those in other countries. For example, OECD countries tend to have fewer self-employed workers as the per capita GDP increases. However, South Korea does not fit this pattern because the income level is higher and the number of self-employed workers is also high. In 2008, one-third of the economically active population in South Korea was self-employed [10]; in 2015, South Korea’s self-employment rate was 25.9%, ranking fifth among all OECD countries and 9.9% higher than the European Union average [27].

Previous studies have shown that precarious employment is associated with various health problems. For example, workers with insecure jobs have higher prevalences of non-communicable diseases such as asthma and coronary heart disease [28, 29]. Non-regular workers also exhibit high frequencies of fatigue, back pain, and musculoskeletal diseases, and they are more likely to be in poor psychological health [30]. These groups of workers also exhibit high mortality from alcohol-related causes and smoking-related cancer, and high overall mortality rates [8]. Self-employed workers have been considered non-standard in some studies [31], and they experience higher levels of job-related stress and more psychosomatic health problems than do those who are not self-employed [32]. Irregular work is associated not only with job insecurity [33] but also with poor on-the-job training [34] and greater exposure to hazardous work conditions [35, 36]. All of these conditions constitute potential psychosocial and material pathways by which health can be damaged [37]. We found disparities in the use of preventative screening by employment precariousness, suggesting that such precariousness may affect health inequities from the early stages of the ill-health spectrum.

Several mechanisms may explain the employment-associated disparities in participation in cancer screening. First, there is a loophole in the system, even though screening is provided as a social service in Korea. According to the National Health Insurance Act and the Occupation Safety Health Act of Korea, all workers who are workplace insured must periodically undergo health screening; office workers must undergo screening every 2 years, and non-office workers, every year [38, 39]. Furthermore, the Occupational Safety Health Act states that business owners who do not facilitate worker health examinations will be fined maximum 10,000 USD (1 USD =1000 Won) [39]. However, most irregular and self-employed workers are regionally insured rather than workplace insured, and thus are not eligible for workplace health checkups. Additionally, individuals who are workplace insured but on short contracts tend to work in the same place for less than a year, making it possible for them to be excluded from screening.

Second, and similar to the above, dispatched or outsourced irregular workers who work for small- or medium-sized companies find it difficult to participate in screening. They represent the most underprivileged workers in South Korea [10]. Whereas workers in large companies have the opportunity to undergo regular screening, workers in small companies may suffer from the owner’s noncompliance with the duty to conduct screening programs. Thus, regular screening may be less accessible to such groups. Indeed, from 2006 to 2013, workers employed by enterprises with < 50 employees were about 50% less likely to participate in general health examinations than were those employed by enterprises with ≥300 employees [40].

Self-employed and non-standard workers may also be unable to take the time off to participate in cancer screening. Self-employed individuals generally tend to operate in workplaces that are smaller than those of wage workers, and they also work long hours. To the extent that the income and job security of this group are similar to those of non-regular workers, self-employed workers also represent an insecure labor force. According to a 2017 report [41], self-employed individuals with no employees comprised approximately 70% of all self-employed persons during 2000–2016 in Korea. Most of these individuals work long hours to make up for the low hourly income and do not take vacations, which can have deleterious effects on health and behavior [42]. Non-standard workers who wish to be screened must ask for permission from the company or take unpaid time off. However, if their jobs are unstable, they would likely not take time off for screening. Indeed, non-standard workers take less sick leave, working while ill instead, because of concerns about job security and the fear of job loss [37], despite the fact that they experience poor working conditions, are treated unfairly, and receive low wages, all of which likely increase the need for healthcare [2, 43].

### Public health implications

We found obvious inequities in the use of preventative services, according to employment conditions. Along the course of the health spectrum, it is possible that a vicious cycle may develop; individuals with precarious work receive fewer preventative services, increasing the incidence of preventable diseases, rendering the economic situation even more difficult. It is necessary for institutions to commit to breaking this cycle. We found that employment conditions per se were independently associated with the use of preventative services, even after controlling for sociodemographic factors such as income and educational level. As employment conditions are associated with wages, occupational safety, and job security, such conditions interact with sociodemographic factors to influence individual health status and the use of health services. However, our findings suggest that relatively simple interventions would allow equitable access to health services, unlike the interventions required to influence other sociodemographic factors.

From the perspective of improving the national cancer screening rate, it is clear that the use of cancer screening services is affected by employment characteristics and insecurity. As mentioned above, biennial gastric cancer screening for those aged ≥40 years is free for Medicaid enrollees and NHI beneficiaries in the lower half of the income strata, and is inexpensive (only 10% of the real cost) for NHI beneficiaries in the upper half of the income strata. This suggests that barriers other than financial barriers exist. Korea has made various efforts to improve cancer screening rates, and the gastric cancer screening rate did increase from 39.2% in 2004 to 73.6% in 2013 [19]. It seems that any improvements in cancer screening rates obtainable via individual encouragement have now been attained. In fact, we found that further adjustment of health-related behavior (which is correlated with individual health commitment) did not significantly change the outcome (Model 3 vs. Model 2 in Table 2). Given that it is necessary to further improve cancer screening rates, a social approach must be added to individual encouragement.

### Limitations

Our study had several possible limitations. First, as the work was cross-sectional in design, it was impossible to determine causal associations between employment conditions and the use of preventative services. Second, both employment status and cancer screening data were self-reported and therefore subject to a degree of inaccuracy. Third, we studied cancer screening only. Further studies are needed to determine whether inequities caused by employment are apparent in the use of other preventative health services such as general health examinations or influenza vaccination. Also, although other cancer screening programs were not included in our study because they did not target the entire population or targeted only older people (reducing the relevance to employment status), it is necessary in the future to investigate whether similar patterns appear when other cancer screening systems are examined. Fourth, although we chose gastric cancer screening to study employees of a wide age range, all ages were not included in the study because eligible age of undergoing gastric cancer screening is over 40 years. In this regard, further investigation of preventive services used by all age groups is needed.

Despite these limitations, our findings are meaningful because the numbers of irregular and the self-employed workers are increasing in Korea, exacerbating health-related problems. Previously, Kim et al. [44] reported a study on cancer screening participation according to job status using 2013 KNHANES data. The study showed that part-time workers were less likely to participate in cancer screening than full-time workers. However, due to a limitation in available survey variables, the study included only wage workers. In our study, we used the 2008–2009 KNHANES data which includes more abundant work-related variables. As a result, we were able to analyze not only wage workers, but also self-employed workers, unpaid family members, and other workers. In addition, we were able to carry out in-depth analysis for wage workers and consider whether they were regular workers (aspects of employment contract term and job security) or dispatched workers (aspects of salary source and business command). To the best of our knowledge, this is the first study to explore disparities in the use of preventative services by various employment characteristics (occupation type, industry, work schedule, and precariousness) and various types of precariousness (work hours, employment contract term, and salary source business command) using nationally representative data.

## Conclusions

We found that employment precariousness was an unrecognized barrier to access to preventative care. Self-employed, irregular, and dispatched workers were significantly less likely to participate in cancer screening than were others. Inequities in the use of preventative services increase overall health inequities. Political efforts should be made to reduce employment insecurity and to improve participation in preventative screening services by vulnerable employees. Otherwise, health inequities will become entrenched.

## Abbreviations

KCDC: Korea Centers for Disease Control and Prevention
KNHANES: Korea National Health and Nutrition Examination Survey
NCSP: National Cancer Screening Program
NHI: National Health Insurance
SEP: Socioconomic Position
UGIS: Upper Gastrontestinal Series
